# Supplementary material for: First Integrative Morphological and Genetic Characterization of Tremoctopus violaceus sensu stricto in the Mediterranean Sea
Source: Animals (Basel). 2021 Dec 30;12(1):80. doi: 10.3390/ani12010080 (PMC8749848; doi:10.3390/ani12010080)
Supplement: Supplementary file 1 [file animals-12-00080-s001.zip › animals-1456188-supplementary.pdf]

Table S1: Details of the COI and 16S sequences analysed in this study

Gene	GenBank Accession	BOLD Accession	Submitted as	Clade	Sampling Location	Reference
COI	OM025233	pending	*Tremoctopus violaceus*	B	Apulia, Italy —Mediterranean Sea	present study
COI	AF377978	GBCPH0220-06	*Tremoctopus violaceus*	A	Florida, U.S.A.—Atlantic Ocean	[55]
COI,16S	KY649286	GBML12075-19	*Tremoctopus violaceus*	A	Taiwan—Pacific Ocean	[56]
COI	MH379648	GBML35597-19	*Tremoctopus gracilis*	A	India—Indian Ocean	Geetha *et al*., unpublished
COI	MN443917	GBMNB71454-20	*Tremoctopus violaceus*	A	South Korea—Pacific Ocean	Kim, unpublished
COI	MW025168	GBMND9913-21	*Tremoctopus violaceus*	A	South Korea—Pacific Ocean	Oh *et al*., unpublished
COI	MK186001	KERCE079-17	*Tremoctopus robsoni*	C	New Zealand—Pacific Ocean	[52]
COI	MN560197	GBMNC27345-20	*Tremoctopus sp.*	C	Chile—Pacific Ocean	[57]
COI	---	CEPHW173-11	Octopoda	A	Japan—Pacific Ocean	Finn & Steinke, unpublished
COI	---	CEPHW176-11	Octopoda	A	Japan—Pacific Ocean	Finn & Steinke, unpublished
16S	OM025092	---	*Tremoctopus violaceus*	B	Sardinia, Italy —Mediterranean Sea	present study
16S	OM025093	---	*Tremoctopus violaceus*	B	Sardinia, Italy —Mediterranean Sea	present study
16S	OM025094	---	*Tremoctopus violaceus*	B	Apulia, Italy —Mediterranean Sea	present study
16S	MT271737	---	*Tremoctopus violaceus*	B	Mexico—Atlantic Ocean	[3]
16S	AJ252767	---	*Tremoctopus violaceus*	A	Hawaii—Pacific Ocean	Hudelot, unpublished
16S	MN435565	---	*Tremoctopus violaceus*	A	South Korea—Pacific Ocean	Kim, unpublished
